# Prevalence of diabetes and presence of autoantibodies against zinc transporter 8 and glutamic decarboxylase at diagnosis and at follow up of Graves’ disease

**DOI:** 10.1007/s12020-019-01852-w

**Published:** 2019-02-19

**Authors:** Berglind Jonsdottir, Ida Jönsson, Mikael Lantz

**Affiliations:** 1 Department of Endocrinology, Lund University, SE-221 00 Lund, Sweden; 2 Department of Clinical Sciences, Skåne University Hospital, SE-205 02 Malmö, Sweden

**Keywords:** Islet autoantibodies, Graves’ disease, Thyroid autoantibodies, Diabetes

## Abstract

**Purpose:**

The aim of this work was to investigate, in patients with newly diagnosed Graves’ disease (GD), the frequency of islet autoantibodies including autoantibodies against Zink transporter 8 (ZnT8A), as well as to investigate the relation between thyroid autoantibodies, islet autoantibodies and diabetes both before GD diagnosis and at follow-up.

**Methods:**

Blood samples from 278 patients with newly diagnosed GD were analyzed for autoantibodies against glutamic acid decarboxylase (GADA), insulinoma-associated protein-2 (IA2-A), three variants of zinc transporter 8 (ZnT8A), thyroid peroxidase (TPOA) and the TSH receptor (TRAb). Information on other autoimmune diseases, as well as development of diabetes during follow up was gathered from patient’s medical journal.

**Results:**

At GD diagnosis, 13.7% were positive for islet autoantibodies, with the majority being positive for GADA (8.7%) and ZnT8A (7.6%). TPOA were found positive in 71% and TRAb in 83%. No association was found between islet autoantibodies and thyroid autoantibodies or diabetes diagnosis during follow up. Positive association was found between islet autoantibodies and all forms of diabetes, diagnosed both before and after GD (OR: 2.5, CI: 1.1–6.8, *p* = 0.03) but not to other autoimmune diseases at GD diagnosis.

**Conclusions:**

The incidence of GADA and ZnT8A in patients with GD is high and might indicate wide range endocrine autoimmunity, as well as risk for non-autoimmune diabetes rather than exclusively mark beta cell autoimmunity and type 1 diabetes.

## Introduction

Graves’ disease (GD) and autoimmune type 1 diabetes (T1D) are common organ specific autoimmune endocrine disorders that may co-occur [1]. T1D is characterized by immune mediated destruction of the pancreatic beta cell, reflected by autoantibodies against glutamic acid decarboxylase (GADA), insulinoma-associated protein-2 (IA2-A), insulin (IAA) and three variants of zinc transporter 8 (ZnT8A) [2, 3]. The predictive value of islet autoantibodies is thoroughly studied and at present well recognized [4, 5], although their pathogenic role is unclear. GD is, in contrast to T1D, characterized by activating autoantibodies to the thyroid hormone stimulation receptor (TRAb). These autoantibodies stimulates thyroid hormone production resulting in thyrotoxicosis [6]. Autoantibodies against thyroid peroxidase (TPOAb) and thyroglobulin (TgAb), can also be found in GD patients, although more characteristic for the other phenotype of autoimmune thyroid disease, Hashimoto’s thyroiditis, often associated with impaired thyroid function.

The co-occurrence of autoimmune endocrine disorders, referred to as autoimmune polyendocrine syndrome (APS), is common in genetic susceptible individuals, where the most common combination is T1D and autoimmune thyroid disease [7].

Thyroid autoimmunity is quite well studied in patients with T1D were 15–30% are reported positive for TPOAb or TgAb [8] while the frequency of islet autoimmunity in patients with GD is less studied with conflicting results on islet cell autoantibodies (ICA) [9, 10].

ZnT8 is the most recent discovered antigen in T1D, interestingly ZnT8 is also found in both thyroid and other endocrine tissue, as well as in adipose tissue [11].

There is a paucity in studies on islet autoimmunity in GD patients and the most recent islet autoantibody, ZnT8A, has not to our knowledge been studied in patients with GD.

The aim of this prospective study was to (1) investigate the frequency of islet autoantibodies including autoantibodies against ZnT8 and to (2) investigate the relation between thyroid autoantibodies and islet autoantibodies, as well as diabetes in patients with newly diagnosed GD. The hypothesis is that islet autoimmunity is common and that ZnT8A are found in patients with newly diagnosed GD as a marker of thyroid or other endocrine autoimmunity, as well as a marker of opthalmopathy as ZnT8 is found in adipose tissue.

## Material and methods

### Study group

Patients living in the town of Malmö Sweden with newly diagnosed thyrotoxicosis due to GD were prospectively registered in the database GD2002 during the years 2002–2011 (*n* = 214) and in the study TT96 1996–2002 (*n* = 64) as previously described [12, 13]. The patients were classified as having GD on the basis of clinical signs, plasma concentrations of TSH <0.2 mIU/L, presence of TRAb and/or a diffuse uptake on technetium scintigraphy. In 2017, samples from 278 patients were available for islet autoantibody analysis. The median follow up time was 9 years (2 months–20 years). Development of diabetes diagnosis, as well as the presence of other autoimmune diseases was gathered from patient records used in clinical routine. The ICD-10 diagnosis E10 and E11 were used for classification of diabetes. Description of the study population is found in Table 1.

The study was approved by the Regional Ethics Review Board in Lund University, Lund, Sweden.

### Assays of thyroid hormones and thyroid autoantibodies

Plasma TSH (reference interval 0.4–3.5 mIU/L, sensitivity 0.001 mIU/L, CV 10%), free T4 (reference interval 8–14 pmol/L, sensitivity 2 pmol/L, CV 10%) and free T3 (reference interval 3.5–5.4 pmol/L, sensitivity 2 pmol/L, CV 10%) were measured with ELISA technique according to the manufacturer’s instructions (Beckman-Coulter). TSH receptor antibodies (TRAb) were measured using a human radioreceptor assay kit purchased from Brahms following the manufacturer’s instructions (reference interval <1 IU/L, sensitivity 0.3 IU/L, CV 9.3–15.4%). The method has been used in clinical routine since April 2004. Previously a second generation TRAb radioreceptor assay was used from Henning Berlin GMBH (18).

Anti-TPO titer was measured with sandwich ELISA technique (Diagnostic Products Corporation) according to the manufacturer’s instructions (normal reference interval <35 kIU/L, sensitivity 5 kIU/L, CV 6%).

The described assays were all used in a clinical routine laboratory at the Department of Clinical Chemistry in Malmö (Table 1).

**Table 1:** Description of the population

Newly diagnosed patients with Graves´ disease	*n* = 278
Median age	*n* = 45 years
Females	*n* = 273 (85.3%)
Males	*n* = 41 (14.7%)
Type 1 Diabetes before GD diagnosis	*n* = 4
Type 2 Diabetes before GD diagnosis	*n* = 6
Median follow-up time	*n* = 9 years
Other autoimmune disease at GD diagnosis	*n* = 15 (5.4%)

### Autoantibodies to GAD65 and IA-2

Recombinant GAD65 and IA-2 were labeled with ^35^S-methionine (PerkinElmer, Waltham, MA, USA) by in vitro coupled transcription and translation using the TNT SP6 coupled reticulocyte lysate system (Promega, Madison, WI, USA) as described [14]. Full length cDNA coding for human GAD65 in the pTNT vector (Promega) (pThGAD65) or the intracellular domain (amino acids 606-979) of IA-2 in the pSP64 Poly(A) vector (Promega) (IA-2ic) were used as templates [15]. GADA and IA-2A were analyzed in a radioligand binding assay (RBA) [14]. Duplicate samples were incubated with radio-labeled antigen. The samples were transferred to filtration plates (Millipore, Solna, Sweden) and IgG antibodies precipitated with Protein A Sepharose (Zymed Laboratories Inc, San Francisco, CA, USA). After washing to remove all unbound antigen supermix scintillation cocktail (Perkin Elmer) was added and the radioactivity counted in a Wallac Microbeta Trilux system (Perkin Elmer). GADA and IA2A levels were expressed as units per mL (U/mL) derived from the WHO standard 97/550. GADA levels >34 U/mL and IA-2A levels >5 U/ml were considered positive.

### Autoantibodies to zinc T8 transporter

Autoantibodies against the three variants of ZnT8, anti-ZnT8 arginine 325 (ZnT8RA), anti-ZnT8 tryptophan 325 (ZnT8WA), and anti-ZnT8 glutamine 325 (ZnT8QA) were analyzed with an RBA as described previously [16]. Duplicate samples were incubated with equal amounts of the three radio-labeled ZnT8 R/W/Q variants. Every sample >59 U/ml was considered positive.

Islet autoantibodies were analyzed at the laboratory at the center for clinical sciences, CRC, Malmö.

### Statistical methods

Statistical analysis was performed using SPSS statistical software (version 25.0, SPSS, Chicago, IL, USA). Differences in proportions between groups were tested using the *χ*^2^ test or Fisher’s exact when appropriated and OR with 95% confidence interval were calculated. The Mann–Whitney *U* test was used to compare two independent groups. *p* < 0.05 was considered significant.

## Results

### Autoantibodies at diagnosis of GD

**Fig. 1:**
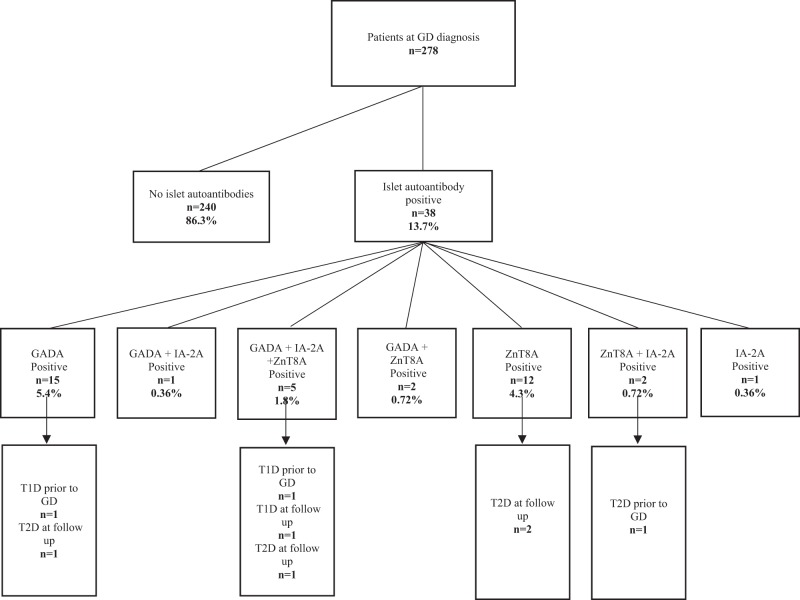
Flow chart of the distribution of islet autoantibodies in the 278 patients with newly diagnosed GD

At the time of diagnosis of GD, 83% (176/212) were TRAb positive. TPOAb analysis at diagnosis was available in 211 patients and of those 150 (71%) were positive. Moreover, 13.7% (38/278) were positive for either GADA, IA-2A, or ZnT8A. The distribution of islet autoantibodies in the population is found in Fig. 1. Taken together, 8.3% (*n* = 23) were positive for GADA, 3.2% (*n* = 9) for IA-2A, and 7.6% (*n* = 21) for ZnT8A.

Patients with positive islet autoantibodies at GD diagnosis were more likely to have any form of diabetes, diagnosed before GD or at follow up (OR 2.7, CI; 1.1–6.8, *p* = 0.03). Otherwise, GD patients with and without islet autoantibodies were similar according to gender, ethnic origin, co-occurrence of another autoimmune disease, smoking and the presence of Graves’ opthalmopathy (Table 2). No significant association was found between islet autoantibodies at diagnosis of GD and development of diabetes during follow up (OR 1.7, CI 0.6–5.6).

**Table 2:** Description of the cohort with and without islet autoantibodies at Graves ‘disease (GD) diagnosis and associations to age, gender, place of birth, other autoimmune disease at diagnosis, smoking, Graves´ opthalmopathy and any form of diabetes diagnosed before Graves ‘disease or at follow up

	GD patients with positive islet Ab (*n* = 38)	GD patients without islet Ab (*n* = 240)	OR	CI 95%	*p* value
Median age (years)	48	45			0.1
Gender	F: *n* = 36 (95%)	F: *n* = 201 (84%)	3.5	0.8–15	0.1
Born in Europe	*n* = 31 (82%)	*n* = 214 (89%)	0.5	0.2–1.3	0.3
Other autoimmune disease	*n* = 2 (5%)	*n* = 13 (5%)	1.7	0.5–5.6	0.3
Diabetes^a^	*n* = 8^b^ (21%)	*n* = 21^c^ (9%)	2.7	1.1–6.8	**0.03**
Smoking	*n* = 17 (45%)	*n* = 100 (42%)	1.1	0.6–2.3	0.4
Graves’ opthalmopathy	*n* = 4 (11%)	*n* = 20 (8%)	1.3	0.4–4.0	0.7

^a^All forms of diabetes, diagnosed before Graves´ disease diagnosis and during follow up

^b^4 were diagnosed before Graves ‘disease (2 with type 1 diabetes). 4 developed diabetes during follow up (1 with type 1 diabetes)

^c^6 were diagnosed before Graves ‘disease (2 with type 1 diabetes)

The bold values are significant

No relation was found between positive TRAb or TPOAb or titers of TRAb or TPOAb, and islet autoantibodies.

### Diabetes in GD at diagnosis and at follow-up

A total of 10 patients had been diagnosed with diabetes prior to GD diagnosis. Of those, 4 had been diagnosed with T1D while 6 had been diagnosed with type 2 diabetes (T2D). Of those diagnosed with type 1 diabetes, one patient was positive for ZnT8A, GADA, and IA-2A, one for GADA and two were islet autoantibody negative at the diagnosis of GD. Two of the 6 patients diagnosed with T2D prior to GD diagnosis were positive for islet autoantibodies at the diagnosis of GD, one for ZnT8A, GADA, and IA-2A and one with ZnT8A and IA-2A. During follow-up, additionally one patient was diagnosed with diabetes and treated with insulin, and 18 with diabetes which was treated with oral medication. The one patient diagnosed with insulin treated diabetes, 6 years after GD debut, was positive for ZnT8A, GADA, and IA-2A at GD diagnosis. Three of the patients diagnosed with diabetes, treated with oral medication, were positive for islet autoantibodies, two for ZnT8A and one for GADA.

**Fig. 2:**
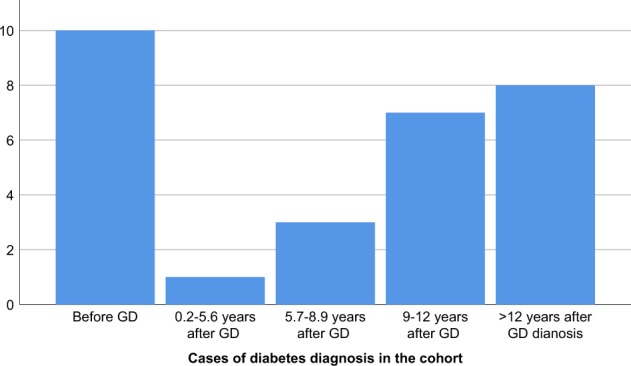
Distribution of diabetes diagnosis in the cohort, both before GD and at follow up

GD patients additionally diagnosed with any form of diabetes (diagnosed before GD or during follow up) were more likely to be islet autoantibody positive at diagnosis of GD (OR: 2.5, CI:1.1–6.8, *p* = 0.03). These patients were also older which is in line with the fact that the majority of diabetes cases occur >5.6 years after diagnosis of GD (Fig. 2). Likewise, 14% of those patients were additionally diagnosed with other autoimmune diseases compared to 4% of the patients not diagnosed with diabetes (*p* = 0.06) (Table 3).

**Table 3:** Description of the cohort with and without diabetes (all types of diabetes, diagnosed before Graves’ disease (GD) and at follow up) and associations to age, gender, place of birth, another autoimmune disease, islet autoantibodies, smoking and Graves´ opthalmopathy

	GD patients with diabetes (*n* = 29)	GD patients without diabetes (*n* = 249)	OR	CI 95%	*p* value
Median age (years)	53.5	43			**<0.001**
Gender	F: *n* = 22 (76%)	F: *n* = 215 (86%)	0.5	0.9–1.2	0.15
Born in Europe	*n* = 28 (97%)	*n* = 218 (88%)	1.8	0.4–8.1	0.3
Another autoimmune disease	*n* = 4 (14%)	*n* = 11 (4%)	3.5	1.0–11	0.06
Islet autoantibodies	*n* = 8 (28%)	*n* = 30 (12%)	2.5	1.1–6.8	**0.03**
Smoking	*n* = 13 (45%)	*n* = 104 (42%)	0.8	0.3–1.4	0.15
Graves’ opthalmopathy	*n* = 3 (10%)	*n* = 21 (8%)	1.3	0.4–1.7	0.4

The bold values are significant

## Discussion

In this study of 278 patients with GD, we found the prevalence of islet autoantibodies to be 13.7% at GD diagnosis with the highest prevalence of GADA and ZnT8A. While the presence of GADA in patients with GD is previously described [9, 10] it was a novel finding that 7.6% of GD patients were positive for ZnT8A at diagnosis. The prevalence of diabetes in our cohort after median follow up time of 9 years was 10% compared to 4.1% in the Swedish population [17]. The high prevalence of islet autoimmunity and diabetes in a population with GD is not unexpected. Although we did not find any relation between islet autoimmunity and development of diabetes in our population, there was a relation between islet autoimmunity at GD diagnosis and diabetes diagnosis, all forms, diagnosed both before GD diagnosis and during follow up. In our cohort, 5 patients diagnosed with T2D (2 prior to GD and 3 at follow up) were actually islet autoantibody positive possibly indicating latent autoimmune diabetes of adulthood, LADA. The fact that most patients were diagnosed with non-insulin dependent diabetes both before and during follow up and the relation found to islet autoimmunity indicate a highly heterogeneous group [18]. It is quite interesting that 9 out of 10 patients with multiple islet autoantibodies at GD diagnosis have not developed diabetes, 6-15 years later. A possible explanation could be that progression to diabetes is slow in this patient category, as described by others [10, 19] which is in line with our finding that the majority of diabetes cases occur >5.6 years after diagnosis of GD (Fig. 2). It would therefore have been interesting to investigate glucose metabolism in the subjects, to identify patients with pre-diabetes or stage 2 autoimmune T1D; positive islet autoantibodies and dysglycemia without symptoms [20], but since this study was performed without continuous collection of plasma samples, it was not possible. In a recent study, first phase insulin response was tested in subjects with GD, finding no significant difference between those with positive autoantibodies for GADA or IA-2A and the ones with negative islet autoantibodies [21]. The islet autoantibodies might not uniquely represent risk for autoimmune T1D, but may instead be an unspecific sign of autoimmune disease.

ZnT8A is the most recent autoantibody in the family of islet autoantibodies utilized in the diagnosis of T1D [22]. ZnT8A have been found related to thyroid autoimmunity in children diagnosed with type 1 diabetes as well in healthy children followed for their increased risk for the disease [23]. Higher titers of ZnT8A have additionally been found to correlate with TPOAb in adults with LADA [24]. Our findings in addition to the above mentioned are of interest considering that ZnT8 is expressed in the thyroid gland, as well as in other endocrine tissues [25]. The existence of ZnT8A might therefore represent a state of endocrine autoimmunity. The role of ZnT8 in the beta cell is well understood were the ZnT8 catalyzes transport of the zinc ion in to the insulin granule were zinc is essential for the processing, storage, secretion and action of insulin [26]. ZnT8 is also expressed in thyroid cells, a clarification of the ZnT8 function in other endocrine cells than beta cells might facilitate understanding of the association between diverse autoimmune endocrine disorders.

We additionally found that 8.3% of the patients were positive for GADA at GD diagnosis, confirming studies in similar cohorts with reported prevalence of 7.2–13% [9, 10, 21]. Interestingly, GADA positivity appear to be more specific for GD than Hashimoto’s thyroiditis when studied in both phenotypes of autoimmune thyroid disease as studies on adult patients diagnosed with either GD or Hashimoto’s thyroiditis find GADA more often in GD patients. In fact, no significant difference was found between GADA prevalence in Hashimoto patients and controls [11, 27]. The autoantigen GAD65 is attached to synaptic vesicles, involved in the synthesis of gamma–aminobutric acid an inhibitory neurotransmitter, in the beta-cells as well as in neurons [28]. No reports are found on the existence of the enzyme in the thyroid gland although various reports are found on GADA positivity and relation to thyroid autoimmunity and autoimmune thyroid disease in patients diagnosed with type 1 diabetes [29, 30]. The appearance of GADA in T1D is found related to both increasing age and HLA DQ 2/DR 3 haplotype [31], a haplotype also found to increase the risk for GD [32]. The presence of GADA and ZnT8A in our cohort hardly indicate the increased risk for type 1 diabetes as only one patient developed insulin treated diabetes during follow-up. We found that islet autoantibodies at GD diagnosis was associated to diabetes, all forms diagnosed both before GD and during follow up, similarly, GADA and ZnT8A were found associated to autoimmune thyroid disease in patients with T2D [33]. The majority of the diabetes diagnosis in our cohort is not autoimmune or insulin dependent why the existence of ZnT8A and GADA in patients with GD is interesting, possibly indicating relation to subgroups of diabetes or/and wide-ranging endocrine autoimmunity due to common genetic susceptibility and/or common environmental triggers. Further prospective studies are needed to investigate the relation between these autoantibodies and progression to different autoimmune endocrine diseases as well as to different forms of diabetes.

In summary we found that 13.7% of the patients were positive for islet autoantibodies at the diagnosis of GD; 8.3% for GADA and 7.6% for ZnT8A–a novel report. No relation was found between positivity to islet autoantibodies and development of diabetes at follow-up nor to thyroid autoantibody positivity or titers. However an association between the presence of islet antibodies and diabetes was found in GD patients. The increased incidence of islet autoantibodies in patients with GD might indicate wide range endocrine autoimmunity and need for further studies on sub classifications of diabetes, particularly in patients diagnosed with other autoimmune disease as GD.
